# Exploring Combined Use of Continuous Glucose Monitoring and Anti‐Diabetes Medications on Glycaemic Control for People With Type 2 Diabetes Not Using Insulin

**DOI:** 10.1002/edm2.70089

**Published:** 2025-08-25

**Authors:** Poorva M. Nemlekar, Katia L. Hannah, Courtney R. Green, Gregory J. Norman

**Affiliations:** ^1^ Health Economics and Outcomes Research, Clinical Affairs Dexcom, Inc. San Diego California USA; ^2^ Medical Affairs Dexcom, Inc. San Diego California USA

**Keywords:** continuous glucose monitoring, diabetes mellitus, GLP‐1 receptor agonist, non‐insulin therapy, type 2

## Abstract

**Introduction:**

Continuous glucose monitoring (CGM) offers a detailed view of glycaemic management, potentially enhancing the effectiveness of non‐insulin, anti‐diabetes medications. This study aimed to evaluate whether CGM use in combination with anti‐diabetes medications is associated with changes in A1c among people with type 2 diabetes not using insulin.

**Materials and Methods:**

This was a retrospective, observational analysis of administrative claims and linked laboratory data from Optum's Clinformatics Data Mart database. The study observation period covered 01/07/2018 through 30/06/2023 with 6‐month baseline and follow‐up periods. CGM use in conjunction with ≥ 1 of five anti‐diabetes medication classes: metformin, sulfonylureas, sodium‐glucose cotransporter‐2 (SGLT2) inhibitors, dipeptidyl peptidase‐4 (DPP‐4) inhibitors and/or glucagon‐like peptide‐1 receptor agonists (GLP‐1 RAs) was required. The primary outcome was change in A1c from baseline. Linear regression models tested the main and interaction effects of CGM and each anti‐diabetes medication.

**Results:**

Overall, 52,394 CGM‐naïve adults with non‐insulin‐treated type 2 diabetes using anti‐diabetes medications were identified (4086 CGM users; 48,308 CGM non‐users). CGM use was associated with a –0.45% greater A1c change among CGM users compared to CGM non‐users (*p* < 0.0001). After adjusting for covariates, CGM users experienced greater A1c reductions vs. CGM non‐users with all medications, but statistically significant interactions showed that for DPP‐4 inhibitors, GLP‐1 RAs and sulfonylureas, there were greater decreases in A1c for CGM users vs. CGM non‐users who were taking the medication compared to CGM users vs. CGM non‐users who were not taking the medication. A1c change between CGM users vs. CGM non‐users did not vary by metformin or SGLT2 inhibitor use.

**Discussion:**

The findings suggest that CGM use could augment the glycaemic benefits of anti‐diabetes medications in people with non‐insulin treated type 2 diabetes. These results support broader adoption of CGM.

## Graphical abstract

In regression models, a significant interaction was found between continuous glucose monitoring (CGM) use and use of 3 anti‐diabetes medication classes: sulfonylureas, DPP‐4 inhibitors and GLP‐1 RAs. This synergistic effect means that CGM could augment the glycaemic benefits of anti‐diabetes medications in people with T2D not using insulin.

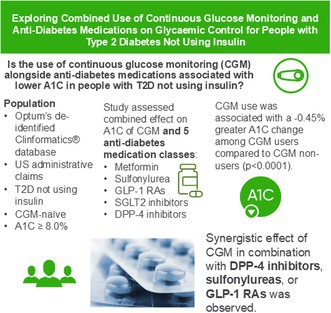

## 1 Introduction

People living with type 2 diabetes are often prescribed multiple non‐insulin anti‐diabetes medications, such as metformin, sulfonylureas, sodium‐glucose cotransporter‐2 (SGLT2) inhibitors, dipeptidyl peptidase‐4 (DPP‐4) inhibitors and glucagon‐like peptide‐1 receptor agonists (GLP‐1 RAs) [1]. Increasingly, many are also using continuous glucose monitoring (CGM) systems to monitor their glucose levels and guide lifestyle modifications [2, 3, 4]. CGM access whether by insurance coverage expansion or the availability of lower‐cost, over‐the‐counter options is expected to increase the number of people with type 2 diabetes using non‐insulin medications and CGM.

CGM provides a more granular view of glycaemic management compared to volitional fingerstick blood glucose or periodic glycated haemoglobin (A1c) measurements. The data from CGM systems can be retrospectively analysed using web‐ or app‐based software, and the results can be interpreted by individual users, their healthcare providers and clinical researchers [5, 6, 7]. Studies have shown that CGM use helps lower A1c among people with type 2 diabetes using intensive insulin therapy (i.e., multiple daily injections or insulin pumps) [2, 8, 9] or basal insulin therapy [2, 9, 10, 11, 12]. Recently, more studies have explored the benefits of CGM among people with type 2 diabetes not using insulin [2, 9, 11, 12, 13].

As the variety of effective medications available for people with type 2 diabetes has reached an all‐time high, we hypothesised that CGM use could provide additional support and help those using anti‐diabetes medications improve their glycaemia. Therefore, we evaluated whether use of CGM in combination with five classes of anti‐diabetes medications was associated with changes in A1c among people with type 2 diabetes not using insulin.

## 2 Materials and Methods

### 2.1 Data Source

This retrospective, observational, database study used de‐identified administrative health claims and linked laboratory data from Optum's Clinformatics Data Mart (CDM) database. The CDM contains de‐identified health information consistent with the Health Insurance Portability and Accountability Act and managed according to Optum customer data use agreements in compliance with Code of Federal Regulations title 45, section 164.514 (b) (1) [14]. CDM administrative claims submitted for payment by HCPs and pharmacies are verified, adjudicated and de‐identified prior to inclusion. These data, including patient‐level enrolment information, are derived from claims submitted for all medical and pharmacy health care services with information related to health care costs and resource utilisation.

### 2.2 Study Design and Cohort Selection

The population included adults ≥ 30 years of age with type 2 diabetes identified using the International Classification of Diseases, 9th/10th Revisions (ICD‐9/ICD‐10) who were enrolled in commercial health plans, including Medicare Advantage health plans. The minimum age of 30 years for study inclusion is consistent with prior studies and is intended to reduce the likelihood of including individuals with type 1 diabetes and/or extensive comorbidities [15, 16]. The study observation period covered 01/07/2018 through 30/06/2023. Type 2 diabetes diagnosis was confirmed with ≥ 1 inpatient hospital or emergency department medical claim or ≥ 2 ambulatory medical claims ≥ 30 and ≤ 730 days apart with an ICD‐9 or ICD‐10 diagnosis code for diabetes in any position on the claim during the study observation period [17].

Individuals with ≥ 1 CGM‐related claim identified through National Drug Codes or Healthcare Common Procedure Coding System codes (Table S1) between 01/01/2019 and 31/12/2022 (study identification period) were included in the analysis. The index date was set as the earliest observed claim for any CGM system, including real‐time CGM and intermittently‐scanned CGM. For the cohort of CGM non‐users, a random index date mirroring the range and distribution of the index dates for CGM initiation in the CGM user cohort was selected. Individuals were required to have continuous health plan enrolment with medical and pharmacy coverage 6 months before (baseline) and 6 months after (follow‐up) the index date. Five classes of anti‐diabetes medications were included: metformin, sulfonylureas, SGLT2 inhibitors, DPP‐4 inhibitors and GLP‐1 RAs (Appendix S1). A person was considered a medication user if there was ≥ 1 pharmacy claim for the medication present during both the baseline and the follow‐up periods. Exclusion criteria included evidence of pregnancy during the study period and claims for a personal CGM before the index date (subjects were CGM‐naïve). Individuals with evidence of basal, bolus or mixed insulin during baseline were excluded. Finally, individuals were required to have ≥ 1 laboratory A1c test result during the baseline period and ≥ 1 A1c laboratory test value during the follow‐up period. A mean baseline A1c ≥ 8.0% was required for study inclusion.

### 2.3 Outcome Measures

The primary outcome measure was change in A1c determined from the average of available A1c values during the baseline and follow‐up periods. Change in A1c was computed as the average A1c follow‐up value minus the average A1c baseline value, with negative values indicating improvement in A1c.

### 2.4 Statistical Analyses

The study specifically assessed whether the effect of CGM use on A1c change varied depending on the use of anti‐diabetes medication. General linear regression models were employed to assess the relationships between the independent variables (CGM use and anti‐diabetes medication use) and the dependent variable (A1c change) controlling for covariates (age, race, gender, insurance type, baseline A1c, Charlson comorbidity score and the presence of other medications). Each model tested the main effect and the interaction effect (i.e., effect modification) of CGM and each anti‐diabetes medication class. Each model also included an interaction term defined as the two‐way interaction between CGM use and anti‐diabetes medication use. Estimated marginal means (e‐means) from the regression models were used to display the A1c change at each level of the two‐way interaction.

## 3 Results

### 3.1 Cohort Characteristics

From a starting population of over 10 million individuals, nearly 4.3 million people were identified as ≥ 30 years of age and living with type 2 diabetes. After applying all inclusion and exclusion criteria, 52,394 people with type 2 diabetes using non‐insulin therapies were identified. Ultimately, 4086 were CGM users and 48,308 were CGM non‐users. Baseline demographic information for each cohort is presented in Table 1. During the follow‐up period, 46.7% of CGM users and 43.3% of CGM non‐users achieved a mean A1c that met the Healthcare Effectiveness Data and Information Set (HEDIS) target of < 8.0% (*p* < 0.0001 between groups).

**TABLE 1:** Baseline demographic and health characteristics.

Characteristic	CGM users	CGM non‐users
*N*	4086	48,308
Age, mean (SD)	67.5 (10.3)	67.0 (10.9)
Male	2130 (52.1)	26,622 (55.1)
Race		
Asian	135 (3.5)	2466 (5.5)
Black	572 (14.8)	6139 (13.6)
Hispanic	438 (11.3)	9524 (21.1)
White	2682 (69.3)	26,332 (58.3)
Unknown	44 (1.1)	705 (1.6)
Missing/not reported	215 (5.3)	3142 (6.5)
Region		
Midwest	788 (19.3)	7649 (15.8)
Northeast	477 (11.7)	4820 (10.0)
South	2138 (52.3)	25,238 (52.2)
West	683 (16.7)	10,591 (21.9)
Baseline A1c, %, mean (SD)	9.46 (1.30)	9.16 (1.18)
Insurance type		
Medicare Advantage	3484 (85.3)	36,982 (76.6)
Commercial	602 (14.7)	11,326 (23.4)
Charlson comorbidity score, mean (SD)	2.02 (1.89)	1.29 (1.55)
Comorbidities		
Chronic kidney disease	1004 (24.6)	6411 (13.3)
Congestive heart failure	579 (14.2)	3691 (7.6)
Diabetic retinopathy	864 (21.1)	4615 (9.6)
Hyperlipidemia	3359 (82.2)	35,712 (73.9)
Hypertension	3337 (81.7)	35,683 (73.9)
Myocardial infarction	257 (6.3)	1590 (3.3)
Obesity	1560 (38.2)	14,242 (29.5)
Peripheral neuropathy	1575 (38.5)	10,217 (21.1)
Medication use		
DPP‐4 inhibitor	180 (4.4)	6582 (13.6)
GLP‐1 RA	657 (16.1)	7633 (15.8)
Metformin	1341 (32.8)	31,946 (66.1)
SGLT2 inhibitor	451 (11.0)	8316 (17.2)
Sulfonylurea	689 (16.9)	21,478 (44.5)

*Note:* Data are *n* (%) unless noted otherwise. A *t* test or chi‐squared test was used to assess statistically significant differences between groups. All comparisons were statistically significant at *p* < 0.01 except GLP‐1 RA use at *p* = 0.66.

Abbreviations: DPP‐4, dipeptidyl peptidase‐4; GLP‐1 RA, glucagon‐like peptide‐1 receptor agonist; SGLT2, sodium‐glucose cotransporter‐2.

### 3.2 Interaction of CGM and Medications

The univariate unadjusted main effect of CGM use was a −0.45% greater A1c change among CGM users compared to CGM non‐users (*p* < 0.0001). After adjusting for covariates, A1c change among CGM users compared to CGM non‐users was attenuated (−0.28%) but remained statistically significant (*p* < 0.0001, Table 2). This A1c change among CGM users was consistent across regression models (−0.26% to −0.27%, all *p* < 0.0001, Tables S2–S6).

**TABLE 2:** Linear regression model without interaction terms with change in A1c as the outcome variable.

Covariate	ΔA1c, B (SE)	95% CI	*p*‐value
(Intercept)	4.89 (0.07)	[4.75, 5.03]	< 0.0001
CGM use	−0.28 (0.02)	[−0.33, −0.23]	< 0.0001
Age at index	0.00 (0.00)	[0.00, 0.00]	< 0.0001
A1c at baseline	−0.60 (0.01)	[−0.61, −0.59]	< 0.0001
Race: Asian^a^	0.04 (0.03)	[−0.01, 0.10]	0.12
Race: Black^a^	0.13 (0.02)	[0.09, 0.16]	< 0.0001
Race: Hispanic^a^	0.18 (0.02)	[0.15, 0.21]	< 0.0001
Race: Unknown^a^	0.14 (0.05)	[0.04, 0.24]	0.004
Insurance type: Medicare Advantage^b^	0.10 (0.02)	[0.06, 0.13]	< 0.0001
Gender: Male	−0.05 (0.01)	[−0.07, −0.02]	0.0002
Charlson comorbidity score at baseline	−0.02 (0.00)	[−0.03, −0.01]	< 0.0001
Metformin use^c^	−0.21 (0.01)	[−0.23, −0.18]	< 0.0001
Sulfonylurea use^c^	0.15 (0.01)	[0.12, 0.17]	< 0.0001
SGLT2 inhibitor use^c^	−0.02 (0.02)	[−0.05, 0.01]	0.22
DPP‐4 inhibitor use^c^	0.04 (0.02)	[0.01, 0.08]	0.02
GLP‐1 RA use^c^	−0.18 (0.02)	[−0.22, −0.15]	< 0.0001

*Note:* ^a^Reference race is White; ^b^reference insurance type is commercial; ^c^defined as use of medication at baseline and follow‐up.

Abbreviations: CGM, continuous glucose monitoring; CI, confidence interval; DPP‐4, dipeptidyl peptidase‐4; GLP‐1 RA, glucagon‐like peptide‐1 receptor agonist; SE, standard error; SGLT2, sodium‐glucose cotransporter‐2.

Mean A1c at baseline was a significant predictor of A1c change at follow‐up. Specifically, each 1.0 percentage point increase in baseline A1c was associated with a 0.6 percentage point decrease in A1c between baseline and follow‐up (*p* < 0.0001, Table 2). Similarly, metformin use (−0.21%) and GLP‐1 RA use (−0.18%) were associated with decreased A1c, whilst sulfonylurea use (0.15%) was associated with increased A1c (all *p* < 0.0001).

Plots of the model‐based e‐means for the two‐way interactions of CGM with each medication class show CGM users experienced greater reductions in A1c (−0.95% to −1.31%) compared to CGM non‐users (−0.69% to −0.89%), regardless of anti‐diabetes medication class use (Figure S1). Statistically significant interaction effects were observed for CGM with three medication classes: sulfonylureas, DPP‐4 inhibitors and GLP‐1 RAs (Tables S2–S4), indicating a greater decrease in A1c for CGM users vs. CGM non‐users who were taking the medication compared to CGM users vs. CGM non‐users who were not taking the medication.

Table 3 shows the e‐means from the regression models for change in A1c between CGM users and CGM non‐users by medication and the corresponding two‐way interaction effect. CGM users experienced a greater decrease in A1c compared to CGM non‐users regardless of sulfonylurea use (Table 3 and Figure S1A). The change in A1c between CGM users and CGM non‐users was greater among sulfonylurea users (−1.10% vs. −0.71%, ΔA1c = −0.39%) compared to those not taking a sulfonylurea (−1.12% vs. −0.86%, ΔA1c = −0.26%), resulting in an estimated interaction effect of β = −0.13 (*p* < 0.05) (Table S2).

**TABLE 3:** Interaction table of estimated model means.

Parameter	CGM users, e‐mean (SE)	CGM non‐users, e‐mean (SE)	Difference in ΔA1c	*p*‐value
Sulfonylurea use	−1.10 (0.06)	−0.71 (0.02)	−0.39	
No sulfonylurea use	−1.12 (0.03)	−0.86 (0.02)	−0.26	
Interaction with CGM			−0.13	0.031
DPP‐4 inhibitor use	−1.31 (0.11)	−0.76 (0.02)	−0.55	
No DPP‐4 inhibitor use	−1.08 (0.03)	−0.81 (0.02)	−0.27	
Interaction with CGM			−0.28	0.009
GLP‐1 RA use	−1.27 (0.06)	−0.87 (0.02)	−0.40	
No GLP‐1 RA use	−0.95 (0.03)	−0.70 (0.02)	−0.25	
Interaction with CGM			−0.14	0.023
Metformin use	−1.20 (0.04)	−0.89 (0.02)	−0.31	
No metformin use	−0.95 (0.03)	−0.69 (0.02)	−0.26	
Interaction with CGM			−0.05	0.35
SGLT2 inhibitor use	−1.15 (0.07)	−0.80 (0.02)	−0.35	
No SGLT2 inhibitor use	−1.05 (0.03)	−0.78 (0.02)	−0.27	
Interaction with CGM			−0.08	0.27

Abbreviations: CGM, continuous glucose monitoring; DPP‐4, dipeptidyl peptidase‐4; GLP‐1 RA, glucagon‐like peptide‐1 receptor agonist; SE, standard error; SGLT2, sodium‐glucose cotransporter‐2.

The association of CGM use with change in A1c varied by DPP‐4 inhibitor use (Table 3 and Figure S1B) where change in A1c between CGM users and CGM non‐users was greater among DPP‐4 inhibitor users (−1.31% vs. −0.76%, ΔA1c = −0.55%) compared to those not taking a DPP‐4 inhibitor (−1.08% vs. −0.81%, ΔA1c = −0.27%) with an interaction effect of β = −0.28 (*p* < 0.01) (Table 3).

Similarly, the association of CGM use with change in A1c varied by GLP‐1 RA use (Table 3 and Figure S1C). Whilst CGM users overall had a greater decrease in A1c compared to CGM non‐users, the change in A1c between CGM users and CGM non‐users was greater among GLP‐1 RA users (−1.27% vs. −0.87%, ΔA1c = −0.40%) compared to those not taking a GLP‐1 RA (−0.95% vs. −0.70%, ΔA1c = −0.25%) (interaction effect β = −0.14, *p* < 0.05) (Table S4).

Whilst there was a numerical difference in A1c change between CGM users and CGM non‐users among metformin users (−1.20% vs. −0.89%, ΔA1c = −0.31%) compared to those not taking metformin (−0.95% vs. −0.69%, ΔA1c = −0.26%), this difference was not statistically significant (β = −0.05, *p* = 0.37, Table 3, Table S5 and Figure S1D).

Similarly, whilst there was a numerical difference in A1c change between CGM users and CGM non‐users among SGLT2 inhibitor users (−1.15% vs. −0.80%, ΔA1c = −0.35%) compared to those not taking SGLT2 inhibitors (−1.05% vs. −0.78%, ΔA1c = −0.27%), this difference was not statistically significant (β = −0.08, *p* = 0.27, Table 3, Table S6 and Figure S1E).

## 4 Discussion

In this retrospective, observational database study of people with type 2 diabetes using anti‐diabetes medications and, in some cases, CGM, use of CGM was associated with greater decreases in A1c compared to CGM non‐use. In addition, the findings suggest there was a synergistic effect of CGM in combination with sulfonylureas, DPP‐4 inhibitors and GLP‐1 RAs.

The observed synergistic effect between CGM use and certain anti‐diabetes medications is notable and, in fact, supports the American Diabetes Association's recent recommendation to consider CGM use among adults with type 2 diabetes treated with glucose‐lowering medications [4]. CGM use alongside anti‐diabetes medications could promote adherence to those medications because users are able to see their glycaemic effects. It is also possible that CGM users shared their data with their healthcare provider, who was able to adjust their medication dosage. Studies have shown that CGM use can reduce therapeutic inertia in clinical practice [18, 19]. CGM use could have also motivated behaviour change [20, 21, 22], which drove additional reductions in A1c over medication use alone.

A consensus statement endorsed by multiple global organisations on the use of CGM in clinical trials [6] underscores its value in measuring the glycaemic impact of medical interventions. The ability to collect glucose data throughout the day and night and analyse an individual's response to food or drink choices, medication taking, physical activity, sleep and many other variables is unmatched. Glycaemic variability and the presence of dangerous hypoglycaemic or hyperglycaemic events can also be assessed with CGM, which A1c measurements, as an average of the previous 3 months of glycaemic control, cannot provide [23].

Compared with other studies of CGM use among people with type 2 diabetes not on insulin therapy, the A1c change described in this study was lower (−0.45% crude or −0.28% after adjusting for covariates) compared to −3.0% in Grace [12] and –1.6% in Wright [11]. However, both of these were single‐arm studies and used real‐time CGM and intermittently scanned CGM, respectively. Our result is in line with the −0.3% adjusted difference in Aronson et al. [13] which compared intermittently scanned CGM use and diabetes education to a control group who received diabetes education alone. In line with our study, these studies similarly required A1c values of ≥ 7.5% [12, 13] or ≥ 8.0% [11]. Finally, a retrospective study of individuals receiving care from an American Medical Group Association (AMGA) member organisation found an A1c decrease of −1.13% among people with type 2 diabetes not on insulin therapy with a baseline A1c ≥ 7.5% [9]. Whilst these comparisons are important to contextualise the findings of this study related to A1c, the aim of this study was to assess the interactions between CGM use and anti‐diabetes medication use.

The synergistic effect described here between CGM and GLP‐1 RA use has been described elsewhere. Wright et al. found that the combined use of CGM and a GLP‐1 RA among people with type 2 diabetes was associated with larger decreases in A1c compared to GLP‐1 RA use alone [24]. Miller et al. described significant A1c improvements among people with suboptimally controlled type 2 diabetes following the addition of CGM to prior GLP‐1 RA therapy [25]. Finally, Ehrhardt et al. and Parsiani et al. [26] described the use of CGM to titrate medications, including GLP‐1 RA therapies, in clinical practice [27].

Strengths of our study include the large study population, the use of a major administrative claims database and the analysis of five different classes of anti‐diabetes medications. Multivariate regression models were adjusted for factors that may influence glycaemia, including baseline A1c and the presence of other anti‐diabetes medications. This allowed us to isolate the combined effect of CGM with each medication class.

There were some notable differences between groups, including a higher proportion of Hispanics, a lower Charlson comorbidity score and higher rates of anti‐diabetes medication use among CGM non‐users, compared to CGM users. These differences, especially the higher rates of CGM non‐use among historically marginalised groups, are the subject of considerable research [28, 29, 30], and these characteristics were all included as covariates in our regression model.

The complexity of type 2 diabetes and its progressive nature may necessitate polypharmacy to achieve an individual's health goals [31]. Whilst our analysis attempted to control for other medication use, the large number of combination medications (Appendix S1), including combinations with medications not assessed in this study (e.g., thiazolidinediones), limited our ability to isolate specific medication effects. It is important to note that the effectiveness of a medication may depend on other factors such as the duration of diabetes and the potency of a medication in the presence of other medications. For example, polypharmacy could have attenuated the glycaemic effects of some medications, including DPP‐4 inhibitors [32] and sulfonylureas.

Other limitations of this study include the all‐US population with a high baseline A1c (≥ 8.0%), which limits generalisability to other countries or more well‐controlled populations. In addition, Optum's CDM database only includes medical claims from commercial insurers (including Medicare Advantage), which could limit generalisability to those on Medicaid or traditional Medicare insurance plans. Whilst our study protocol was not prospectively registered, this retrospective, observational study was exploratory in nature. The study period included the COVID‐19 pandemic, which could have influenced both medication taking and lifestyle factors like food intake or physical activity. In addition, some medications, particularly GLP‐1 RAs, were subject to shortages during our study period [33], which could have impacted user adherence to their medication. Medication or CGM adherence can impact therapy effectiveness [34, 35]; however, user adherence to therapies was not assessed in this study. Our analyses were limited to medical and pharmacy claims with linked laboratory data, which do not include potentially important information such as duration of diabetes, health awareness or educational level. We did not consider the presence of other non‐diabetes medications or differentiate anti‐diabetes medications by strength or dosage. For example, our study included multiple available GLP‐1 RAs (Appendix S1), which could have significantly different impacts on A1c.

## 5 Conclusions

These findings suggest that CGM may be effective for improving glycaemic control among people with type 2 diabetes not using insulin when used alone or in combination with anti‐diabetes medications. Greater access to CGM may help more people learn about their diabetes, the medications they are prescribed and their role in managing their health status.

## Author Contributions

**P.M.N.:** conceptualisation; data curation; formal analysis; methodology; visualisation; writing – review and editing. **K.L.H.:** conceptualisation; methodology; writing – review and editing. **C.R.G.:** visualisation; writing – original draft preparation; writing – review and editing. **G.J.N.:** conceptualisation; methodology; supervision; visualisation; writing – review and editing. All authors approved the final version of the manuscript.

## Disclosure

Prior Presentation: Portions of this article were presented at the 84th Scientific Sessions of the American Diabetes Association held in Orlando, Florida on 20–23 June 2024.

## Conflicts of Interest

All authors are employees of Dexcom Inc.

## Supporting information


**Appendix S1:** National drug codes for anti‐diabetes medications.

**Figure S1:** Change in A1c by CGM use and use of anti‐diabetes medication.

**Table S1:** National drug codes (NDCs) and healthcare common procedure coding system (HCPCS) codes for CGM systems included in the analysis.
**Table S2:** Linear regression model including the two‐way interaction term for CGM use and sulfonylurea use.
**Table S3:** Linear regression model including the two‐way interaction term for CGM use and DPP‐4 inhibitor use.
**Table S4:** Linear regression model including the two‐way interaction term for CGM use and GLP‐1 RA use.
**Table S5:** Linear regression model including the two‐way interaction term for CGM use and metformin use.
**Table S6:** Linear regression model including the two‐way interaction term for CGM use and SGLT2 inhibitor use.

## Data Availability

The data that support the findings of this study are available from Optum. Restrictions apply to the availability of these data, which were used under license for this study. Data are available from the authors with the permission of Optum.
