# Supplementary figures and images for: Exploring Combined Use of Continuous Glucose Monitoring and Anti‐Diabetes Medications on Glycaemic Control for People With Type 2 Diabetes Not Using Insulin
Source: Endocrinol Diabetes Metab. 2025 Aug 25;8(5):e70089. doi: 10.1002/edm2.70089 (PMC12375899; doi:10.1002/edm2.70089)

**A**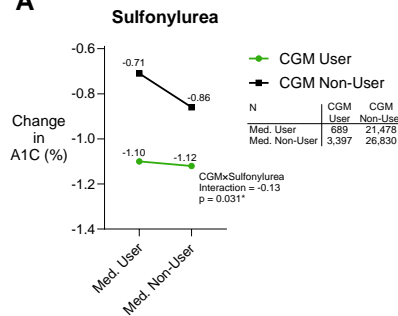**B**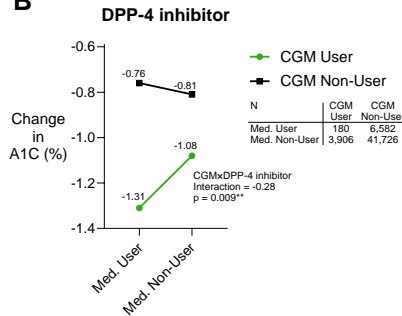**C**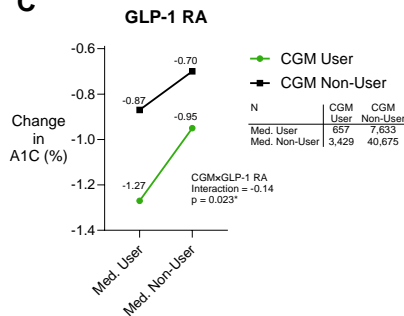**D**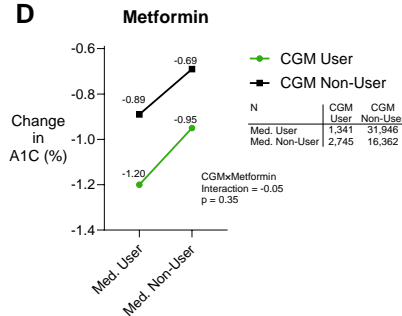**E**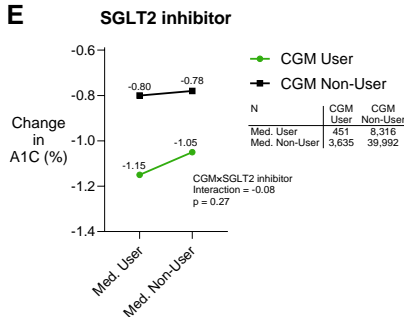

**Supp. Fig. 1. Change in A1c by CGM use and use of anti-diabetes medication**

Supplement: Supplementary file 2 — Figure S1: Change in A1c by CGM use and use of anti‐diabetes medication. [file EDM2-8-e70089-s003.pdf]
