# Supplementary material for: Exploring Combined Use of Continuous Glucose Monitoring and Anti‐Diabetes Medications on Glycaemic Control for People With Type 2 Diabetes Not Using Insulin
Source: Endocrinol Diabetes Metab. 2025 Aug 25;8(5):e70089. doi: 10.1002/edm2.70089 (PMC12375899; doi:10.1002/edm2.70089)
Supplement: Supplementary file 3 — Table S1: National drug codes (NDCs) and healthcare common procedure coding system (HCPCS) codes for CGM systems included in the analysis. Table S2: Linear regression model including the two‐way interaction term for CGM use and sulfonylurea use. Table S3: Linear regression model including the two‐way interaction term for CGM use and DPP‐4 inhibitor use. Table S4: Linear regression model including the two‐way interaction term for CGM use and GLP‐1 RA use. Table S5: Linear regression model including the two‐way interaction term for CGM use and metformin use. Table S6: Linear regression model including the two‐way interaction term for CGM use and SGLT2 inhibitor use. [file EDM2-8-e70089-s001.docx]

# Supplementary Tables

**Supplementary Table 1**. National drug codes (NDCs) and healthcare common procedure coding system (HCPCS) codes for CGM systems included in the analysis

| **CGM** | **HCPCS and NDC Codes** |
| --- | --- |
| **HCPCS** | A4226, A4239, A9276, A9277, A9278, E2103, K0553, K0554, S1030, S1031, S1034, |
| **Abbott Freestyle Libre** | 57599000019, 57599000021, 57599000101, 57599000200, 57599080000, 57599080300, 57599081800 |
| **Dexcom** | 08627000301, 08627001011, 08627001021, 08627001031, 08627001101, 08627001301, 08627001401, 08627001601, 08627002011, 08627002021, 08627002031, 08627003011, 08627003021, 08627003031, 08627004104, 08627005011, 08627005021, 08627005031, 08627005104, 08627005303, 08627006011, 08627006021, 08627006031, 08627008011, 08627008021, 08627008031, 08627009011, 08627009111 |
| **Medtronic** | 43169070405, 43169075272, 43169080040, 43169094604, 43169095568, 63000017962, 63000028585, 63000028677, 63000028678, 63000031699, 63000033698, 63000035751, 63000035844, 63000041434, 63000041435, 65781045101, 65781045111, 65781045121, 65781045141, 65781045151, 76300000211, 76300000214, 76300000260, 76300000610, 76300000701, 76300000805, 76300001101, 76300001701, 76300007201, 76300007202, 76300010001, 76300010002, 76300017962, 76300023982, 76300027842, 76300040001, 76300055111, 76300055113, 76300055114, 76300055115, 76300070501, 76300070601, 76300070701, 76300072501, 76300075111, 76300075112, 76300075113, 76300075114, 76300075115, 76300285852, 76300286774, 76300286781, 76300720301, 76300731001, 76300731501, 76300750001, 76300751001, 76300770001, 76300790001 |
| **Eversense** | 17491002145, 17491002235 |
| **Unknown** | 17491002142 |

**Supplementary Table 2**. Linear regression model including the two-way interaction term for CGM use and sulfonylurea use.

| **Covariate** | **ΔA1c**  **β (SE)** | **95% CI** | **p-value** |
| --- | --- | --- | --- |
| (Intercept) | **4.89 (0.07)** | [4.74, 5.03] | <0.0001 |
| CGM use | **-0.26 (0.03)** | [-0.31, -0.21] | <0.0001 |
| Age at index | **0.00 (0.00)** | [0.00, 0.00] | <0.0001 |
| A1c at baseline | **-0.60 (0.01)** | [-0.61, -0.59] | <0.0001 |
| Race: Asian* | 0.04 (0.03) | [-0.01, 0.10] | 0.11 |
| Race: Black* | **0.13 (0.02)** | [0.09, 0.16] | <0.0001 |
| Race: Hispanic* | **0.18 (0.02)** | [0.15, 0.21] | <0.0001 |
| Race: Unknown* | **0.14 (0.05)** | [0.05, 0.24] | 0.004 |
| Insurance type: Medicare Advantage^ | **0.09 (0.02)** | [0.06, 0.13] | <0.0001 |
| Gender: Male | **-0.05 (0.01)** | [-0.07, -0.02] | 0.0002 |
| Charlson comorbidity score at baseline | **-0.02 (0.00)** | [-0.03, -0.01] | <0.0001 |
| Metformin use^†^ | **-0.21 (0.01)** | [-0.23, -0.18] | <0.0001 |
| Sulfonylurea use^†^ | **0.15 (0.01)** | [0.13, 0.18] | <0.0001 |
| SGLT2 inhibitor use^†^ | -0.02 (0.02) | [-0.05, 0.01] | 0.24 |
| DPP-4 inhibitor use^†^ | **0.04 (0.02)** | [0.01, 0.08] | 0.02 |
| GLP-1 RA use^†^ | **-0.18 (0.02)** | [-0.22, -0.15] | <0.0001 |
| Interaction: CGM use × sulfonylurea use | **-0.13 (0.06)** | [-0.25, -0.01] | 0.03 |

*Reference race is White; ^Reference insurance type is Commercial; ^†^Defined as use of medication at baseline and follow-up

Abbreviations: CGM: continuous glucose monitoring; CI: confidence interval; DPP-4: dipeptidyl peptidase-4; GLP-1 RA: glucagon-like peptide-1 receptor agonist; n.s.: not statistically significant; SE: standard error; SGLT2: sodium-glucose cotransporter-2

**Supplementary Table 3**. Linear regression model including the two-way interaction term for CGM use and DPP-4 inhibitor use.

| **Covariate** | **ΔA1c**  **β (SE)** | **95% CI** | **p-value** |
| --- | --- | --- | --- |
| (Intercept) | **4.89 (0.07)** | [4.74, 5.03] | <0.0001 |
| CGM use | **-0.27 (0.02)** | [-0.31, -0.22] | <0.0001 |
| Age at index | **0.00 (0.00)** | [0.00, 0.00] | 0.0001 |
| A1c at baseline | **-0.60 (0.01)** | [-0.61, -0.58] | <0.0001 |
| Race: Asian* | 0.04 (0.03) | [-0.01, 0.10] | 0.12 |
| Race: Black* | **0.13 (0.02)** | [0.09, 0.16] | <0.0001 |
| Race: Hispanic* | **0.18 (0.02)** | [0.15, 0.21] | <0.0001 |
| Race: Unknown* | **0.14 (0.05)** | [0.05, 0.24] | 0.004 |
| Insurance type: Medicare Advantage^ | **0.10 (0.02)** | [0.06, 0.13] | <0.0001 |
| Gender: Male | **-0.05 (0.01)** | [-0.07, -0.02] | 0.0002 |
| Charlson comorbidity score at baseline | **-0.02 (0.00)** | [-0.03, -0.01] | <0.0001 |
| Metformin use^†^ | **-0.21 (0.01)** | [-0.23, -0.18] | <0.0001 |
| Sulfonylurea use^†^ | **0.15 (0.01)** | [0.12, 0.17] | <0.0001 |
| SGLT2 inhibitor use^†^ | -0.02 (0.02) | [-0.05, 0.01] | 0.23 |
| DPP-4 inhibitor use^†^ | **0.05 (0.02)** | [0.01, 0.09] | 0.008 |
| GLP-1 RA use^†^ | **-0.18 (0.02)** | [-0.22, -0.15] | <0.0001 |
| Interaction: CGM use × DPP-4 inhibitor use | **-0.28 (0.11)** | [-0.50, -0.07] | 0.009 |

*Reference race is White; ^Reference insurance type is Commercial; ^†^Defined as use of medication at baseline and follow-up

Abbreviations: CGM: continuous glucose monitoring; CI: confidence interval; DPP-4: dipeptidyl peptidase-4; GLP-1 RA: glucagon-like peptide-1 receptor agonist; n.s.: not statistically significant; SE: standard error; SGLT2: sodium-glucose cotransporter-2

**Supplementary Table 4**. Linear regression model including the two-way interaction term for CGM use and GLP-1 RA use.

| **Covariate** | **ΔA1c**  **β (SE)** | **95% CI** | **p-value** |
| --- | --- | --- | --- |
| (Intercept) | **4.89 (0.07)** | [4.74, 5.03] | <0.0001 |
| CGM use | **-0.26 (0.03)** | [-0.31, -0.21] | <0.0001 |
| Age at index | **0.00 (0.00)** | [0.00, 0.00] | 0.0001 |
| A1c at baseline | **-0.60 (0.01)** | [-0.61, -0.58] | <0.0001 |
| Race: Asian* | 0.04 (0.03) | [-0.01, 0.10] | 0.11 |
| Race: Black* | **0.13 (0.02)** | [0.09, 0.16] | <0.0001 |
| Race: Hispanic* | **0.18 (0.02)** | [0.15, 0.21] | <0.0001 |
| Race: Unknown* | **0.14 (0.05)** | [0.05, 0.24] | 0.004 |
| Insurance type: Medicare Advantage^ | **0.10 (0.02)** | [0.06, 0.13] | <0.0001 |
| Gender: Male | **-0.05 (0.01)** | [-0.07, -0.02] | 0.0002 |
| Charlson comorbidity score at baseline | **-0.02 (0.00)** | [-0.03, -0.01] | <0.0001 |
| Metformin use^†^ | **-0.21 (0.01)** | [-0.23, -0.18] | <0.0001 |
| Sulfonylurea use^†^ | **0.15 (0.01)** | [0.12, 0.17] | <0.0001 |
| SGLT2 inhibitor use^†^ | -0.02 (0.02) | [-0.05, 0.01] | 0.23 |
| DPP-4 inhibitor use^†^ | **0.04 (0.02)** | [0.01, 0.08] | 0.02 |
| GLP-1 RA use^†^ | **-0.17 (0.02)** | [-0.21, -0.14] | <0.0001 |
| Interaction: CGM use × GLP-1 RA use | **-0.14 (0.06)** | [-0.26, -0.02] | 0.02 |

*Reference race is White; ^Reference insurance type is Commercial; ^†^Defined as use of medication at baseline and follow-up

Abbreviations: CGM: continuous glucose monitoring; CI: confidence interval; DPP-4: dipeptidyl peptidase-4; GLP-1 RA: glucagon-like peptide-1 receptor agonist; n.s.: not statistically significant; SE: standard error; SGLT2: sodium-glucose cotransporter-2

**Supplementary Table 5**. Linear regression model including the two-way interaction term for CGM use and metformin use.

| **Covariate** | **ΔA1c**  **β (SE)** | **95% CI** | **p-value** |
| --- | --- | --- | --- |
| (Intercept) | **4.89 (0.07)** | [4.75, 5.03] | <0.0001 |
| CGM use | **-0.26 (0.03)** | [-0.32, -0.21] | <0.0001 |
| Age at index | **0.00 (0.00)** | [0.00, 0.00] | <0.0001 |
| A1c at baseline | **-0.60 (0.01)** | [-0.61, -0.58] | <0.0001 |
| Race: Asian* | 0.04 (0.03) | [-0.01, 0.10] | 0.12 |
| Race: Black* | **0.13 (0.02)** | [0.09, 0.16] | <0.0001 |
| Race: Hispanic* | **0.18 (0.02)** | [0.15, 0.21] | <0.0001 |
| Race: Unknown* | **0.14 (0.05)** | [0.05, 0.24] | 0.004 |
| Insurance type: Medicare Advantage^ | **0.10 (0.02)** | [0.06, 0.13] | <0.0001 |
| Gender: Male | **-0.05 (0.01)** | [-0.07, -0.02] | 0.0002 |
| Charlson comorbidity score at baseline | **-0.02 (0.00)** | [-0.03, -0.01] | <0.0001 |
| Metformin use^†^ | **-0.20 (0.01)** | [-0.23, -0.18] | <0.0001 |
| Sulfonylurea use^†^ | **0.15 (0.01)** | [0.12, 0.17] | <0.0001 |
| SGLT2 inhibitor use^†^ | -0.02 (0.02) | [-0.05, 0.01] | 0.23 |
| DPP-4 inhibitor use^†^ | **0.04 (0.02)** | [0.01, 0.08] | 0.02 |
| GLP-1 RA use^†^ | **-0.18 (0.02)** | [-0.22, -0.15] | <0.0001 |
| Interaction: CGM use × metformin use | -0.05 (0.05) | [-0.14, 0.05] | 0.35 |

*Reference race is White; ^Reference insurance type is Commercial; ^†^Defined as use of medication at baseline and follow-up

Abbreviations: CGM: continuous glucose monitoring; CI: confidence interval; DPP-4: dipeptidyl peptidase-4; GLP-1 RA: glucagon-like peptide-1 receptor agonist; n.s.: not statistically significant; SE: standard error; SGLT2: sodium-glucose cotransporter-2

**Supplementary Table 6**. Linear regression model including the two-way interaction term for CGM use and SGLT2 inhibitor use.

| **Covariate** | **ΔA1c**  **β (SE)** | **95% CI** | **p-value** |
| --- | --- | --- | --- |
| (Intercept) | **4.89 (0.07)** | [4.75, 5.03] | <0.0001 |
| CGM use | **-0.27 (0.03)** | [-0.32, -0.22] | <0.0001 |
| Age at index | **0.00 (0.00)** | [0.00, 0.00] | <0.0001 |
| A1c at baseline | **-0.60 (0.01)** | [-0.61, -0.58] | <0.0001 |
| Race: Asian* | 0.04 (0.03) | [-0.01, 0.10] | 0.12 |
| Race: Black* | **0.13 (0.02)** | [0.09, 0.16] | <0.0001 |
| Race: Hispanic* | **0.18 (0.02)** | [0.15, 0.21] | <0.0001 |
| Race: Unknown* | **0.14 (0.05)** | [0.05, 0.24] | 0.004 |
| Insurance type: Medicare Advantage^ | **0.10 (0.02)** | [0.06, 0.13] | <0.0001 |
| Gender: Male | **-0.05 (0.01)** | [-0.07, -0.02] | 0.0002 |
| Charlson comorbidity score at baseline | **-0.02 (0.00)** | [-0.03, -0.01] | <0.0001 |
| Metformin use^†^ | **-0.21 (0.01)** | [-0.23, -0.18] | <0.0001 |
| Sulfonylurea use^†^ | **0.15 (0.01)** | [0.12, 0.17] | <0.0001 |
| SGLT2 inhibitor use^†^ | -0.02 (0.02) | [-0.05, 0.02] | 0.35 |
| DPP-4 inhibitor use^†^ | **0.04 (0.02)** | [0.01, 0.08] | 0.02 |
| GLP-1 RA use^†^ | **-0.18 (0.02)** | [-0.22, -0.15] | <0.0001 |
| Interaction: CGM use × SGLT2 inhibitor use | -0.08 (0.07) | [-0.22, 0.06] | 0.27 |

*Reference race is White; ^Reference insurance type is Commercial; ^†^Defined as use of medication at baseline and follow-up

Abbreviations: CGM: continuous glucose monitoring; CI: confidence interval; DPP-4: dipeptidyl peptidase-4; GLP-1 RA: glucagon-like peptide-1 receptor agonist; n.s.: not statistically significant; SE: standard error; SGLT2: sodium-glucose cotransporter-2
